# Extra-Limites

**Published:** 1839-04-01

**Authors:** 


### EXTRA-LIMITES.

### Biographical Memoir and Portrait of Dr. James Johnson, Senior Editor of the Medico-Chirurgical Review.*

"Nec medici, nec imperatores, nec oratores, quamvis artis præcepta perceperint, quidquam magnæ laudis dignum sine usu, et exercitatione consequi possunt."—Cicero.

A justly celebrated physiologist and physician† has well observed, that "that man is a scientific physician, who is well acquainted with, and has appropriated to his own use, the results of all the inquiries which have been made at different times by eminent observers upon the symptoms, course, and causes of diseases, and with the precepts of treatment which they have recommended and employed." And that, "to become a skilful practitioner, he must understand how to bring this knowledge into operation, and be ready to apply all its rules and deductions to each particular case." This is a talent only to be acquired by the most patient observation, the most diligent study, and the most extensive practice; and if any one member of the medical profession is to be regarded as having most zealously laboured to attain this summit of medical knowledge, it is the subject of the present memoir, who has now for many years given to us the fullest evidence of his erudition and practical knowledge by the judicious and able manner in which he has conducted the "Medico-Chirurgical Review, and Journal of Practical Medicine," a work which must be admitted to hold the very first rank and importance in medical periodical literature.

The opinion thus directly given of the merits and qualifications of Dr. James Johnson is not simply a result derived from the perusal of the pages of his review; but has been formed from an acquaintance with his practice, and an association with him in professional matters at various times, and during several years. It is not to be regarded as the language of panegyric—it is truly the payment of a debt of gratitude to one who has so ably led the minds of the medical public to a consideration of what is due to themselves as practitioners, and to those individuals whose happiness and lives have been committed to their care. The importance of an able and just periodical literature of medical science must be admitted by all—it is too generally entrusted to inexperienced hands, and crude theory has but too often usurped the place of practical observation. The press of the present day teems with the productions of authors on medical subjects, and literary discrimination is more necessary than ever, to point out not only to the student, but also to the practitioner, the works of real value and necessary to be perused. This is a task of no little labour or difficulty—great information is necessary on the part of the guide, to accomplish this object in a satisfactory manner. Pope observed, that "the greater part of critics form a general character from the observation of particular errors, taken in their own oblique or imperfect views; which is as unjust, as to make a judgment of the beauty of a man's body from the shadow it happens to cast in such or such a position." As a critic, Dr. Johnson is not open to this censure—he stands indubitably one of the least prejudiced, and the manly way in which he has at

* By the kindness of Mr. Pettigrew and Messrs. Fisher and Son, we have been favoured with 2500 impressions of the Portrait herewith presented to the subscribers of this Journal, and the Memoir is copied from the 12th part of the Medical Portrait Gallery.

† Tiedemann—Physiologie des Menschen.

all times stated his objections to the opinions expressed by others, shows that he does not belong to the class so forcibly described by the poet just quoted.

Dr. JAMES JOHNSON was born in the year 1778, in the parish of Ballinderry, county of Derry, Ireland, on the banks of Lough Neagh. His parents were Protestants; his father, a respectable yeoman, cultivating a small farm of thirty or forty acres. James Johnson was the youngest son of a large family, none of whom, I believe, except himself, are now living. At the age of six years he was put to a grammar-school, kept by a Catholic, the brother of the parish priest. Here he made rapid progress, and, as I learn, was generally at the head of his class. When not so, he was very unhappy, and would sit up till midnight in study. At the early age of fifteen he passed an examination in Dublin, in the classics, and was apprenticed to a surgeon-apothecary (Mr. Young) in Port-glenone, in the county of Antrim. He remained there only two years, when he was transferred to Mr. Bankhead of Belfast, where he continued two years more, and then came to London, without either money or friends. He became assistant to an apothecary in the metropolis, and, by hard study and irregular attendance on lectures in anatomy and surgery, he passed a creditable examination at Surgeons' Hall in 1798, and was appointed surgeon's mate in the navy, in the month of May of the same year. In the Mercury frigate, he sailed to Newfoundland and Nova Scotia, always studying very hard, and, when the ship was in harbour, taking every opportunity of visiting the naval hospitals, abroad and at home. Captain Rogers, of the Mercury, who had a great antipathy to the Irish, made an exception in the case of his youthful surgeon's mate, and winked at his absence from the ship for some months in the Winter of 1799, when he studied night and day in London; and in January, 1800, he passed a triumphant examination, for the second time, at Surgeons' Hall. Through the interest of his patron, Captain Rogers, he was appointed full surgeon in the navy, and appointed to the Cynthia, sloop-of-war, on the 27th of February, 1800, as appears by Steel's Navy List. He was then in the twenty-second year of his age. In this ship he accompanied the famous expedition to Egypt, was at the siege of Belleisle, (not the American Belleisle,) and all the various descents which the troops made on the coasts of France, Spain, &c. till they reached Egypt. In the Mediterranean he was taken ill, and was sent back to Gibraltar Hospital, where he did duty for some time, under Mr. Vaughan, surgeon of the Naval Hospital there. From thence he returned to London in the Winter of 1800, and studied in Great Windmill Street, under Mr. Wilson and Mr. Thomas. In the Winter of that year, he distinguished himself as a dissector, and very generally prepared the subjects for Mr. Wilson's and Mr. Thomas's lectures and demonstrations, as the latter gentleman still states with pleasure. It was in this Winter that the present Master of the Rolls, (then Mr. Bickersteth,) and Mr. Johnson formed a society of six individuals, who gave demonstrations daily, in their turn, to a large class of medical students, in the anatomical theatre of Windmill Street.

In May, 1801, Mr. Johnson's slender finances were entirely exhausted, having expended his last farthing on lectures and studies. In the Spring of that year, being anxious to attend a course of midwifery lectures, but not having the means of paying the fee, he stated his circumstances to the late Dr. John Clarke, then a distinguished lecturer in Burlington Street, who instantly gave him a free ticket of admission, and invited him to his table. Mr. Johnson never forgot this act of generosity, and has frequently related the anecdote.

In June, 1801, Mr. Johnson applied to the Navy Medical Board for a ship, and tendered a certificate from Mr. Wilson, couched in the following remarkable terse language to Dr. Harness :—" The *bearer* of this, Mr. James Johnson, has actually *lived* in the dissecting-room of Great Windmill Street during the last six months. Examine him, and see whether he has studied in vain." Dr. Harness instantly appointed him to the " Driver" sloop-of-war, in which ship he served in the North Sea, visiting the Orkney and Shetland Islands, and going with a convoy to the vicinity of Greenland and Hudson's Bay.

At the peace of 1802, he was again out of employ, and passed a few months, and spent the remainder of his scanty finances in study in the metropolis. It then required great interest to get employment on the peace-establishment, and Mr. Johnson had none. He applied to the Sick and Hurt Board, and the late Sir Gilbert Blane having entered into conversation with Mr. Johnson, gave him a help, and he was immediately appointed to a fine frigate, (the "Caroline,") fitting for the East Indies. In May, 1803, he sailed for the East; and during the next three years, in India and China, he laid the foundation for his first medical work, *The Influence of Tropical Climates on European Constitutions.*

In 1806 he returned from the East Indies, and having been successful in prize-money, he now entered as a student at Guy's and St. Thomas's, and became acquainted with Sir Astley Cooper, Dr. Curry, and other distinguished characters of the period. In the Autumn of the same year he married Miss Charlotte Wolfenden, of Lambeg, in the county of Antrim; and after a year or two of attendance on prisoners-of-war at Plymouth and Portsmouth, he was appointed to the "Valiant," of seventy-four guns, in which ship he remained nearly five years, and saw a great deal of active service. This was one of the two line-of-battle ships that forced their way into Basque Roads, between strong batteries, and burnt the French fleet there. Afterwards, in 1809, he was present at the Walcheren expedition, and narrowly watched the havoc of disease on those pestiferous islands.

In 1812, he published the first edition of his work on *Tropical Climates,* chiefly at his own risk and expense, and immediately on its appearance, he was appointed flag-surgeon, with the late Sir William Young, then in command of the North Sea fleet. Here he did the duty of physician to the fleet, and acquired the friendship and patronage of Admiral Young, which continued till the death of the latter in 1820.

At the peace of 1814, the late King, then Duke of Clarence, hoisted his flag in the "Impregnable," when Sir William Young retired, and Mr. Johnson was so strongly recommended to the Duke, that he was retained, and served with His Royal Highness while conveying the Emperor of Russia, King of Prussia, &c. &c., to this country. The Duke had an attack of his hay-asthma at Boulogne, while waiting for the crowned heads, and Mr. Johnson attended His Royal Highness, and the attack was very soon overcome. The Duke was so much pleased with Mr. Johnson, that he exerted all his influence to obtain for him the rank of physician to the fleet, but was baffled by Lord Melville, then at the head of the Admiralty. The Duke appointed him surgeon-in-ordinary, and always afterwards treated him with great kindness.

At the conclusion of the war in 1814, Mr. Johnson settled at Portsmouth as a general practitioner, and in less than two years got into extensive practice. But his health was not good; and perhaps his ambition was strong, for in July, 1818, he removed to London. This was a bold step. With the exception of Sir William Young, he had no friends whatever in the metropolis: he had a family of five children; and, as he has told me, was not worth five hundred pounds in the world. Since that period—now twenty years or more—his life, in a professional point of view, has become well known. He had taken out a Scotch degree in 1813; and he became a licentiate of the College of Physicians in 1820.

Dr. Johnson's first publication was not a medical one. It was entitled *The Oriental Voyager*, published in 1808, and presented an amusing account of his voyages and observations in the East. His next was the work *On Tropical Climates*, published in 1812, and which has gone through five editions. The most experienced practitioners in the diseases of hot climates have uniformly expressed the high opinion they entertain of this work. The application of physiological knowledge to the treatment of disease is beautifully illustrated by the author. The fifth edition contains the substance of his treatise on Indigestion.

While serving with Sir William Young in the North Sea, he published some papers in the *New Medical and Physical Journal;* and when he settled at Portsmouth, he appeared as one of the editors of that work. The Journal, however, had but little success; and in July, 1818, when Dr. J. came to the metropolis, he took the bold and dangerous step of starting a *Quarterly Review,* entirely at his own risk and expense, and conducted by himself alone! To his astonishment, the first edition of the first number was exhausted in the first week, and the work rapidly rose to a circulation of 1250, 1500, 2000, and ultimately 2500 copies. This journal is the only medical one that has ever been reprinted in a foreign country. It has been republished regularly in New York for many years past, and circulates widely in the United States. His private practice increased with the Journal, and the mental and corporeal labour required for his public and professional avocations was enormous, and such as would have destroyed the health of any one who had not an excellent original constitution, and great facility of composition, verifying what the best prose writer perhaps of the present day has observed, that "he who thinks least about it when engaged in composition will be most likely to attain it, for no man ever attained it by labouring for it."* I know, on the best authority, that for years and years Dr. Johnson never even read over the copy of his reviews before it went to press; and so few were the corrections afterwards made, that the cost of these seldom exceeded a few shillings on each quarterly number of his journal. Dr. J. has often declared, that the only secret of his being able to go through such extensive literary labour was his punctuality. Whatever might be his professional avocations of the day, he seldom or never went to bed till the number of pages necessary for the Journal were completed. When private practice was not pressing, he took care to have the Journal far in advance, so that it was always ready long before quarter day. Excepting when on his tours of health, he never relaxed an hour, or hardly a minute, during the day, from work of some kind or other. Indeed, his excursions at home and abroad were not even exceptions to this law; for incessant activity of mind and body has been the characteristic of his life.

Such uninterrupted labour, however, could not be carried on for years with entire impunity. In 1823, after suffering severely from a surgical operation, he recruited his health by a three months' tour in Switzerland, &c.; but in 1826 his dyspeptic complaints assumed an aggravated form, and threatened his life. At this period he was obliged to relinquish, for a time, his professional avocations, and in that retirement wrote his *Essay on Indigestion,* drawn from personal as well as general observations on that afflicting and Proteian malady, the scourge of those who overwork the brain as well as the body. This work, which has gone through nine editions, and has been translated and reprinted in different countries, brought his private practice to the highest point compatible with his health, which of late years has been remarkably good. The first three editions of this work were demanded in the short space of nine months. Few books upon a subject which has been so generally treated of, and upon diseases with which so many are afflicted, have been so popular, yet so entirely devoid of quackery. Beaumont and Fletcher have truly said,

> "What an excellent thing did God bestow on man
> When he did give him a good stomach."

Dr. Johnson felt the want of this blessing, and applied his mind to the relief of

* The Doctor, Vol. II. p. 201.

his sufferings—this has contributed to the happiness of others, for the treatment he proposes is at once energetic, and founded upon a due observation of the phenomena of disease, and the operations of the animal economy. The last edition of the work has a description of the Baths of Pfeffers in the country of the Grisons.

In 1831 he published his first edition of *Change of Air, or the Pursuit of Health*, which has gone through four editions, and is considered by himself as the best of his literary labours, though apparently written *currente calamo*. This volume was the result of an autumnal excursion through France, Switzerland, and Italy, in the year 1829, and contains many judicious observations on the moral, physical, and medicinal influence of travelling, exercise, change of scene, foreign skies, and voluntary expatriation. The work opens with reflections on education and avocation, and most truly depicts the "wear and tear" of human life. This applies equally to the body and the mind, the connexion between which and their sympathies, in all the modifications of pleasure and of pain, and the relation which obtains between the condition of the intellectual faculties and those functions which constitute the animal economy, are well known to be subjects of great intricacy and difficulty. They belong properly, perhaps, more to the natural philosopher than to the moralist, but the researches of either have hitherto not been productive of any positive information upon the subject. The alliance, however, must be admitted—the connexion is apparent, though the cause be obscure. All the great writers of antiquity have remarked upon the effects of excess on the operations of the intellect. Horace devotes a satire to the advantages of temperance, and he remarks, with that energy which so particularly distinguishes all his writings, that the body overcharged with the excess of yesterday, weighs down the mind together with itself, and fixes to the earth that particle of the divine spirit.

" ———— Vides ut pallidus omnis

Cœnâ desurgat dubiâ; quin corpus onustum

Hesternis vitiis, animum quoque prægravat unâ,

Atque affigit humo divinæ particulam auræ."—Hor. Sat. ii. l. 2.

No one has, in my opinion, more tersely or more truly expressed the value of health than Sir William Temple:—

"Health (he says) is the soul that animates all enjoyments of life, which fade and are tasteless, if not dead, without it: a man starves at the best and the greatest tables, makes faces at the noblest and most delicate wines, is old and impotent in seraglios of the most sparkling beauties, poor and wretched in the midst of the greatest treasures and fortunes: with common diseases strength grows decrepit, youth loses all vigour, and beauty all charms; music grows harsh, and conversation disagreeable; palaces are prisons, or of equal confinement; riches are useless, honour and attendance are cumbersome, and crowns themselves are a burden; but, if diseases are painful and violent, they equal all conditions of life, make no difference between a prince and a beggar; and a fit of the stone or the colic puts a king to the rack, and makes him as miserable as he can do the meanest, the worst, and most criminal of his subjects."

Dr. Johnson not only points out the cause of the "Wear and Tear of Modern Life," but he distinctly states the means of counteracting these effects; and all who delight in the union of literary taste with scientific inquiry will peruse Dr. J.'s work with great satisfaction.

In 1833, he published an amusing tour to the Hebrides, entitled *The Recess, or Autumnal Relaxation in the Highlands and Lowlands.*

In 1836, he published *The Economy of Health, or Stream of Human Life*, which has gone through three editions, and is a very popular production. Butler says,

"There is a kind of physiognomy in the titles of books, no less than in the faces of men, by which a skilful observer will as well know what to expect from the one as the other."

Here is matter for the metaphysician and the moralist, as well as the physician. The stream of life from the cradle to the grave!

> " ———————— so gliding on
> It glimmers like a meteor, and is gone!" ROGERS.

And 'tis what Shakespeare said of love—

> " The uncertain glory of an April day,
> Which now shows all the beauty of the sun,
> And by and by a cloud takes all away."

For, as Felltham has written,

" The life of man is the incessant walk of time, wherein every moment is a step towards death. Even our growing to perfection is a progress to decay. Every thought we have is a sand running out of the glass of life. Every letter, which I now write is something cut off from the measure of my existence here."

Dr. Johnson divides it into ten septenniads, and treats of all its various conditions—the evolution and progress of functions—the changes peculiar to the different periods—the diseases of most common occurrence, under varieties and vicissitudes of climate and seasons—and the gradual decay of the mortal fabric. All these important matters are the subjects of Dr. J.'s philosophical observation and speculation, and the manner in which they are treated illustrates the advantages arising from a comprehensive knowledge of the whole science.

During all this time his literary labours in the *Medico-Chirurgical Review* have been indefatigable, though assisted by his son, and by various writers now employed in that work. For the first ten or twelve years, almost every article in that journal was written by himself, for which I have his own testimony, affording a sufficient proof of the assiduity of his studies, and the remarkable facility of his compositions. He states himself to have been almost entirely *self-taught*, both literary and professional; and from the expiration of his short apprenticeship, he supported himself without ever receiving a shilling from his relations.

Through a long and chequered life, he seems to have offered an exception to the dictum of the poet:—

> " Haud facile emergunt, quorum virtutibus obstat
> Res Angusta Domi"

for he overcame all obstacles apparently without difficulty, and rose to comparative affluence and reputation, by easy but regular exertion of his intellect. Considering the difficulty and danger of the office of reviewer, I believe that he has made exceedingly few personal enemies—and most of these few have become his friends in the sequel.

In private practice he is one of the most popular physicians of this metropolis. His manners are mild and kind to his patients, and he has the art of inspiring great confidence in those whom he attends—an art, which like that of poetry,

> " Nascitur non fit."

In his domestic affairs he has been fortunate and happy. His eldest son, Mr. H. J. Johnson, is united with his father as editor of the review, and is very much liked as a teacher of anatomy in the Kinnerton-Street School, and bids fair to arrive at lucrative and honourable distinction in his profession. His second son Mr. W. Johnson, took honours at Cambridge, obtained a fellowship, and is called to the bar. His third son is a solicitor: and his youngest son is studying under his eldest brother for the profession, at St. George's Hospital. His only daughter is married to a gentleman in the legal profession. Dr. Johnson may, therefore, now be considered as practising for the love of his profession, rather than for the support of a family, who are almost all provided for. He has always been a sedulous attendant on the various medical societies, and an active promoter of medical discussions in these institutions, where, indeed, he seems to be a general favourite.—Though remarkably cheerful in society, I have reason to believe that the subject of this memoir is pensive and rather melancholic in private. This is probably the case with a majority of those whose literary productions and convivial conversations would lead us to think them the gayest of the gay. In religion, general politics, and medical politics, Dr. J. is known to be liberal, though free from scepticism, or ultra-radicalism. In the relations of private and domestic life, nothing is known but what is most honourable to his character.

---

### DELIRIUM TREMENS.

Our readers must all remember a very unseemly TRIAL of a septuagenarian surgeon of a public institution, for mistaking delirium tremens, or delirium traumaticum for mania. Now we would ask the hypercritics in such cases, whether mania does or does not exist in fully-formed delirium tremens? No man who ever saw the disease would attempt to deny the existence of mental derangement in the case under consideration. Why, in the United States, where the disease is ten times more prevalent than in this country, it is termed "MANIA A POTU." But the sticklers for a definite mark of diagnosis, say, "Oh we can tell delirium tremens from common mania by its *cause*, not by its SYMPTOMS." Indeed! This is rather an unscientific procedure as well as a very unsafe one. We are called to a patient, and asked to examine him and give the name of his malady. But after a close investigation, we say—"Oh you must tell us the *cause* of his complaint, otherwise we cannot tell you its name." This is a precious piece of diagnosis! Then let us observe that, in the first place, delirium tremens does *not* always acknowledge intemperance for its cause—and that the ordinary cases of acknowledged mania do very frequently result from habits of intoxication. Thus a man who lives well, but not intemperately, meets with a compound fracture of the leg, and consequently is debarred from animal food and wine. He gets delirium tremens or traumaticum, and presents the same phenomena as the man who has been drunk every day. An internal inflammation, as pneumonia, will sometimes occasion all the symptoms of delirium tremens, in good constitutions, but where food and wine are withdrawn, and the lancet and purgation employed. Thus, then, in a majority of cases, the *symptoms* are precisely those of a sudden burst of mania—often taking on the character even of monomania—and the *causes* are often those of common mania, and by no means universally intemperance in spirituous or vinous potations. We have seen several instances of temporary mania assuming the character of delirium tremens so exactly, that nothing but the history of the cases undeceived us. We may mention a recent case. A young medical gentleman was suddenly taken with symptoms of delirium tremens, and we were sent for to visit him. He had red ferretty eyes—cold clammy skin—dry tongue—incessant jactitation—quick small pulse—no sleep for some nights—violent gesticulations—rapid talking—aberration of intellect. He averred that the devil and a coati mondi were under the bed, and that conspiracies were formed against his life. There was tremor of the hands and agitation of the limbs. In short, a more complete picture of delirium tremens we had never seen—and this we mentioned to one of his friends, while we inquired whether or not he had been lately indulging in drink. We were informed that he was a "Tea-totaller"—a young man of the most temperate habits—but that some moral causes, of a very exciting nature, had lately been in active operation, the precise nature of which we do not deem it necessary to mention. This information modified our treatment, or rather our prognosis. We did not exhibit opium or stimulants, but prescribed soothing and sedative remedies—few of which, however, were taken. In ten or fourteen days he was well. A remarkable occurrence took place during our attendance on this gentleman. A female acquaintance much interested in his fate, visited him and remained several hours at his bed-side. The state of her friend's case made such an impression on her nervous system, that, soon after her return home, she presented a train of symptoms resembling those already described—merely from sympathy. We did not ourselves see the lady after she left our patient's bed-side; but were credibly assured of the above occurrence. Here then we see the effects of purely moral causes—namely, the symptoms of delirium tremens.

The advocates of a definite line between delirium tremens and a sudden outburst of mania have still a strong-hold to retreat to, if beaten from the outworks. The treatment, they say, is different in the one case from that which is proper in the other. On this point we have no fear of joining issue with them. In the first place, what line of distinction can be drawn in the treatment where, as is often the case, the mental derangement, acknowledged as such, results from habits of intemperance? None.—But whatever be the cause of the mental aberration, the essential moral treatment is in all cases the same. The patient must be kept under surveillance—or even under restraint if necessary. In delirium a potu, the tendency to self-destruction is often as strong as in mania from any other cause. The utility and necessity, therefore, of personal restraint is as necessary in the one case as in the other, whenever the aberration is considerable, and violence is manifested.

Thus, then, we find an identity of symptoms—often an identity of cause—and frequently a similarity of treatment in mania "*a potu,*" and mania from any other cause. It is hardly necessary to observe that the adjunct "tremens" is obviously improper, since it is by no means a necessary accompaniment of mania "*a potu.*" It is often present in other complaints, or even where no complaint is made by the patient, but where habits of intemperance are established.

If the above observations are correct, or founded on observation, (and we believe they are)—if mania a potu cannot always be distinguished by its symptoms or its causes from any other case of mania—and if the moral treatment, as far as restraint is concerned, be the same in both—then we say that Sir Anthony Carlisle has been hardly treated for mistaking a case of delirium tremens or traumaticum for one of mental derangement. The great ground of complaint against Sir A. has been the order to remove the patient from the ward of an hospital to an asylum. Now we confess that we do not see clearly the justice of this complaint. We conceive that, during the existence of the delirium or mental aberration, no place can be worse, either for the patient himself, or the other inmates of the institution, than the ward of an hospital. The room, or even the dark cell of an asylum is infinitely preferable during the temporary insanity.

We allude only to the diagnosis of delirium tremens and temporary mental derangement, in this case. If the medical officer was guilty of negligence in his duty, the charge should have been placed on its proper basis, and no side wind ought to have been taken advantage of, to bring forward an accusation of ignorance.

### Some Cases by Dr. Beemner.

### Case of Introsusceptio cured by forcing Air into the Intestines.

James Thompson, æt. 44, of a rather spare habit of body, but in the general enjoyment of good health, was suddenly taken ill with pain in his bowels, about ten o'clock in the evening of the 25th Nov. last. He took some spirits, warm drinks, bathed his feet in warm water, and applied warm fomentations to the belly, thinking the pain would wear off. It continued to increase however, and I saw him on the morning of the 26th. I found him labouring under the following symptoms. Pulse full and not particularly hurried—tongue clean—face anxious—belly not distended, and partially relieved by severe but equitable pressure. No alvine discharge since 4 p. m. the preceding day—pain about the umbilicus most excruciating—not steady, but at intervals of from three to four minutes, accompanied by severe vomiting and great thirst. I immediately ordered a purgative glyster, which came off almost immediately, bringing with it the contents of the rectum without abating the pain. I ordered its repetition—part returned the same as thrown up, and part remained; but the violent pain still continued. I then applied a blister over the whole surface of the abdomen, and gave him a powerful opiate; after an hour this settled the vomiting, and in some degree dulled the pain. I then gave him s. m. hyd. gr. xii. and left him. Twelve hours after the pain and vomiting had returned as violently as ever—still no pain on pressure. I then endeavoured to open the bowels by throwing up as much warm water by the domestic machine as I possibly could; he complained of straitness, and part of the water returned without any effect—I then repeated the calomel and left him. On the morning of the 27th, the calomel had proved useless, and the vomiting and pain were as violent as ever; thirst severe, and perspiration profuse. On pressing the belly pain was now felt in the region of the caput cæcum. It now was evident that unless he was speedily relieved death must ensue. I again attempted to overcome the obstruction by throwing up about a gallon of warm water until it was forced back and a considerable part discharged without the least relief. Having some little time ago seen the injection of air suggested in cases similar (I think in a recent Number of the Medico-Chirurgical Review, although I cannot lay my hand on it just now), I determined to try it. Having nothing at hand but a common bellows, I inserted the tube into the rectum, and commenced gradually to force up the cold air. As soon as it found its way into the intestines, the patient said he felt somewhat easier, and I persevered until I could force no more. In a second or two the rarified air was forced back with great violence bringing with it the remaining portion of the water I injected, but nothing more. He said he felt rather easier and urged its repetition. This I did for other two different times with no appearance of relief. On the fourth trial, however, the room was filled with a most fetid smell and a very free discharge of fæculent matter—he felt relieved, the vomiting ceased, and he complained only of general soreness. I now gave him 8 grs. of cal. with one grain p. opii, and left him. That evening he had an alvine discharge, and on the following morning he got ol. ricini, ℥j. which operated freely in the course of the day, and the cure was complete.

Whether the foregoing case was real introsusceptio or not I am not prepared to say, but I am sure of this, that, had I not succeeded by the forcing up of air into the intestines, there were no other means that I am aware of whereby I had the least chance to save the poor fellow. I have given the case at this length, as I conceive the plan pursued worthy the attention of the profession in similar cases. It would be absurd to draw a conclusion from a single case, but from what occurred to me in this instance, the practice appears to be perfectly harmless, since the moment the air became rarified it came off with great force, and left no disagreeable consequence behind.

### POST MORTEM EXAMINATION OF A CASE OF DROPSY.

Mrs. Rae, æt. 68, applied to me about the end of Nov. last; she complained of difficulty of breathing. I found her emaciated—pale—pulse weak—tongue dry and morbidly red—urine scanty—belly swelled and tense—fluctuation distinctly felt. She had been married, had several abortions, but never bore a child at the full period. As the swelling of the abdomen was not sufficient to account for the great difficulty of breathing, on examination I had every reason to think there was water in the chest; from this and her general broken down state of health, I did not consider myself justifiable in drawing off the water, but ordered a few of the pil hyd. with a mixture of squills and digit. and made her drink freely of the sup. tart. pot. in gruel without any beneficial effects—she expired on the 2d Dec.. On laying open the chest the right lung appeared healthy, with scarcely any water in that pleural cavity. The left lung was greatly diminished in volume, the cavity being completely filled with urine. The heart and aorta were healthy, with scarcely any water in the pericardium. The abdominal cavity was completely filled with serum, but not particularly distended. The abdominal viscera generally presented nothing peculiar from ordinary cases of dropsy, but those on the pelvis were one mass of disease. The bladder was much enlarged, and adhered in the greater part of its posterior surface to the left ovary, which was distended much in the form of a pear, the upper portion pointing towards the umbilicus, its diameter at the middle was nearly four inches, and its long diameter about six and a half. To its posterior surface in its whole extent the rectum firmly adhered, and was with difficulty dissected off. On laying the tumor open, it proved to be a complete cyst filled with serum, and the bottom of this cyst was a congeries of smaller ones, varying from the size of a pin's head to that of a pea, presenting the appearance of a half-expanded cauliflower. The uterus was so contracted and embedded in the mass that it was with difficulty distinguished. The right ovary was about the size of a walnut, much of the same shape and filled with a soft substance exactly resembling brown paint. The bladder contained a good many loose particles of sand, some of considerable size, and more than two-thirds of its whole extent were covered with a calcareous deposit, not in lines or in one particular place, but in patches, varying in size from an eighth to two inches in diameter. When these patches were taken out, the scalpel would not pass through them, and they broke over like a piece of mica; when touched on a yielding surface by a metallic body they gave a sensation like that the sound does in striking a stone. The woman to my knowledge never had a symptom of calculus, or complained of any disease of the urinary organs.

In this case, there were many loose particles of sand, and those of sufficient size, had they passed into the urethra in an awkward manner, to have called attention to the bladder, and had the sound been used a stone would have been indicated. Had an operation been undertaken, no knife would have passed through the neck of the bladder had it been considered necessary to cut that part, as it was completely surrounded by calcareous deposit. But independent of this it is possible that, in similar states of the bladder, an operation might be undertaken, and still no stone there. The only thing valuable in this case is the extent of the deposit and its disposition, which may direct the attention of others to diseases of a similar nature.

JAMES BEEMNER, M.D.

*Huntly, N. B. Dec. 5, 1838.*

### Remarkable State of Disease in a Physician.

Dr. R——m, 64—ailing for ten years—for first five years, restlessness or fidgets—could not sleep but a few minutes in one position—increasing in corpulence during that time, though he could take very little food. Only took two glasses of Sherry for drink. About five years ago began to refer to the sigmoid flexure of the colon as the seat of uneasiness. Would take no medicine, but trusted to abstinence for relief. Referred to the region of the duodenum, in which he fancied there was a kind of intussusception. He felt pain in that part. He suspected also that he had hydrothorax. Severe head-aches and dry retching after the least indulgence in wine—say, three or four glasses. In the month of February 1838, complained of cough, difficulty of breathing, depression of spirits, and inability to use exertion of any kind.

Last illness—beginning of September removed to Aldbro', very ill. Pain in the chest so great as to impede the breathing—increased pain in the colon. Took active purgatives and colchicum there. About the 6th of September, began to shew symptoms of jaundice. Frequent retching—could only keep down some strong coffee, or soda water. The limbs now began to emaciate, but the body retained its embonpoint. The jaundice daily increased, till the skin was a deep copper-colour. The urine became like clear port wine—pulse never varied from 70 to 75. About the 10th of September, and before he took the above medicines, he passed spontaneously a strange sort of motion, without smell or colour. He was freely leeched, and the leeches bled copiously. Had repeated hæmorrhages from the bowels, of a dark melenic character. Never could sleep more than a few minutes at a time, for a month before his death. Believed that he had scirrhus of the colon for years—but did not draw the attention of the medical gentlemen to this till about a week before his death, when Mr. Evans detected an induration in the angle made by the transverse arch and descending colon. This induration rolled about and eluded the grasp. These symptoms went on increasing till death, which seemed to take place more from inanition than from the disease. He was latterly supported entirely by beef-tea *per anum*. He died on the 11th of October, 1838.

### Dissection twelve hours after Death.

Skin of a deep copper-colour, approaching to mahogany. The body was not emaciated; but the limbs were much attenuated. The integuments of the abdomen were loaded with fat; and this was deposited in great quantities throughout every part of the abdomen. Stomach collapsed—large intestines distended with air—liver very much exsanguious, and exhibiting the granulated structure clearly. From each cut a glairy, greenish, nearly transparent fluid oozed out.

Gall-bladder presented a contracted appearance. Its coats were very much thickened, and it contained only about a dessert-spoonful of bile, resembling paste in consistence. The ductus cysticus, hepaticus, and part of the ductus communis were completely obliterated by the pressure of a cluster of surrounding glandular bodies, and by an indurated pancreas, forming a large and dense mass around the ducts in question, and completely annihilating all communication between the liver and the duodenum. Stomach, duodenum, ileum and cæcum healthy. The colon was healthy till near its termination in the rectum, where there was a circumscribed ulcer about the size of a crown-piece, at which place the gut was so contracted as only to admit the ring-finger. The ulcer presented slightly thickened and everted edges, and the mucous and muscular coats of the intestine having been destroyed, leaving the peritoneal covering only entire. The pancreas was enlarged and indurated, completely surrounding the bile-ducts, and obliterating their canals. The right kidney was enlarged, and the ureter distended with a green fluid resembling cystic bile. As the ureter approached the bladder, however, it became so contracted as scarcely to leave any passage for the urine. The substance of this and the other kidney did not present any material change of structure, but on the convex surface of each was a cyst filled with transparent fluid—one of them as large as a nutmeg. The head and chest were not examined. (Signed) F. BELL.

The foregoing case of our brother practitioner is not a little remarkable, and will furnish the reader with several subjects for reflection. Not the least curious phenomenon in the history is the long continuance of that restlessness at night, called the "FIDGETS," without any local or tangible cause for such uneasiness. It is tolerably clear, however, that lesions, of various kinds, were going on for many years, in some important organs of the abdomen. We could get but a very imperfect history of the case—and that entirely from the memory of a friend of the deceased, so that perhaps a more minute detail of the symptoms would have developed some interesting symptoms in this complication of maladies. The preservation of such quantities of fat in the abdomen, while the upper and lower extremities were like those of a skeleton, is curious. It is sufficiently evident that the bile-ducts must have been obliterated for a considerable time, and this renders the fact of the obesity still more curious, since theory, experiment, and observation had taught us that, when no bile passed into the duodenum, emaciation was an invariable consequence.

---

### REPORT OF THE MEDICAL AND SURGICAL SOCIETY OE NEWCASTLE-UPON-TYNE,* ON THE PRESENT STATE OF THE MEDICAL PROFESSION.

THIS Society, on surveying the condition of the Medical Profession, cannot but be strongly impressed with the varied and incongruous materials of which it is composed; they find its members, notwithstanding the perfect identity of their avocations, consisting of Practitioners authorised by many different Colleges and Corporate institutions, from whom have been required unequal courses of study, and dissimilar examinations into the extent of their professional and scientific attainments. They discover also that these institutions have been found inadequate to the fulfilment of their intended purposes; that, consequently, persons are engaged in practice, who have not been authorised by any of them, and that a numerous class of individuals are allowed to prey upon the community, under pretence of having become acquainted with operations and modes of curing diseases, unknown to those who have endeavoured, by an appropriate education, to qualify themselves for the treatment of the various infirmities and injuries with which the human frame is liable to be afflicted.

Public safety demands that all persons engaged in the practice of Medicine and Surgery should be duly qualified, by a previous course of study, to discharge the important duties they are called on to perform; and this Society deem it incumbent on the Legislature to provide, that no person shall be permitted to enter upon the execution of those duties, who has not been properly tested as to his acquirements, and publicly declared to possess the requisite degree of qualification.

In consequence of the imperfection and defective administration of the laws at

---

* The Society consists of more than fifty members.

present in existence, they have afforded no security to the public, and have yielded no adequate protection to the legally-authorised practitioner, against an unfair and unjust competition with individuals possessing neither license nor diploma, and who are unable to produce satisfactory evidence of their having devoted sufficient time or attention to the attainment of medical and surgical knowledge.

Quackery, of every description, is allowed to exert its baneful influence on society; and, while its miraculous achievements are continually paraded before the public eye, thousands of its credulous and unsuspecting victims are daily submitting themselves to the pain and torture of secret processes of treatment. It is to be deplored that any part of the public revenue should be derived from the indirect encouragement of a system replete with fraud, and productive of most disastrous consequences.

Chemists and druggists are in the constant habit of prescribing for diseases, with whose nature they can be very imperfectly acquainted.

The Corporate Bodies presiding over the profession appear to be quite unequal to the correction of these abuses. The London College of Physicians have at all times acted on a system of exclusiveness and monopoly, and have never displayed the slightest anxiety for the well-being of the profession at large. The charter of the College of Surgeons confers upon that corporation no control over persons practising surgery; submission to their examinations and bye-laws being entirely *voluntary*. The Company of Apothecaries have, by Act of Parliament, power to prosecute all persons practising as Apothecaries without their license. They have, however, exercised this power in a very limited number of instances, nor do they possess the means of exerting it in a manner likely to counteract the magnitude of the evils in question.

Medical education requires considerable amendment. The long space of time occupied in the apprenticeship of most general practitioners is usually passed in a very unprofitable manner. Apprenticeships are objectionable in many respects and are totally inefficient as a medium of professional improvement; hence, after their termination, a course of study requires to be entered upon, which must, of necessity, be compressed into a period by far too limited for the attainment of the object in view. The information acquired is almost inevitably of a superficial character, for, in addition to hospital practice and dissections, the student is compelled to attend several lectures daily on a variety of subjects; from all of these it is impossible that he can derive full advantage. Clinical instruction is much neglected, and the student completes his prescribed curriculum with a very inadequate preparation for the practical duties awaiting him at the bed-side of the sick.

Certificates of attendance on lectures and hospital practice are frequently granted without enquiry as to the student's diligence, and in some instances are surreptitiously obtained.

The examinations for licences and diplomas are conducted not unfrequently in a cursory and inefficient manner. They do not afford an opportunity of ascertaining the *practical* acquirements of the candidate, nor the extent to which his knowledge is the result of *clinical* observation and experience. The College of Surgeons in London, require certificates of candidates, having studied the various departments of medical science, but their examinations are confined to anatomy, physiology, and surgery. The Society of Apothecaries take no cognizance of surgery, therefore their licentiates being legally qualified, are at liberty to practise *every* branch of the profession, without having been examined in surgery, or required to produce evidence of having been engaged in the study of that important subject.

There are in the United Kingdom of Great Britain and Ireland, not fewer than nineteen Corporations having power to grant degrees in medicine and surgery, and differing essentially in the extent and duration of the curricula they enjoin.

The Society is of opinion that all of these might be advantageously superseded by one institution being placed at the head of the profession in each division of the Empire, whose privileges should be reciprocal, and whose executive officers, elected by the members at large, should hold periodical conferences, for the purpose of establishing uniformity of operation ; that such Institutions should have entire control over education, and the granting of degrees and licenses to practise, together with all other matters relating to the medical profession ; that the course of instruction and test of qualification should be the same in each ; that from one or other of them, all persons engaged in the practice of medicine and surgery, should be required to possess a diploma or license ; that, to individuals thus authorized, the law should extend a suitable protection, and that proper measures should be enforced for the suppression of unqualified practitioners.

The Society would suggest, as a means of effecting the last-named object, that every person before commencing practice in any town or other locality, should be required to obtain a certificate from a magistrate giving him permission to that effect, which should be granted on the production of satisfactory testimonials of qualification, and that, after having been thus authorized, his name should be duly registered. Persons presuming to practise, whose names have not been so registered, should be subjected to a penalty on summary conviction before a justice of the peace.

The rapid progress of science during the present century, in conjunction with increased facilities for the attainment of medical and surgical knowledge, have fully proved, that any attempt to constitute an arbitrary division of diseases, and to consign the treatment of them to different classes of practitioners, according as they affect the external or internal parts of the body, is not only unscientific but impracticable ; and, as the physician and the surgeon must be guided by similar principles in combating disease, whether involving the surface or the interior of the human frame, the education of all practitioners should, in the opinion of this Society, be regulated by one common standard. Those distinctions in rank which have hitherto subsisted (not perhaps without good effect) would thus be rendered unnecessary, since there could be no longer any rational ground for separating into different grades, men who would be identified not less in education, than in the nature and object of their pursuits. Such uniformity, if established in this as *in other countries*, would place practitioners, whether of medicine or of surgery, on an equal footing ; but would not, in the least degree, prevent individuals devoting their energies to the prosecution of any particular department of professional duty, which inclination or other circumstances might lead them to adopt in preference to another.

The task of preparing the medicines, prescribed by the general practitioner, devolves, almost universally, on the apprentice of the latter. That material benefit might accrue from a well-devised scheme of pupilage, there can be no question ; but apprenticeships, as at present conducted, have ever been productive of unhappy results ; and in no respect does the unfavourable tendency of this system appear more conspicuous, than when viewed as an instrument for executing the responsible duty of dispensing. The abolition of apprenticeships, so far, at least, as this department is concerned, would be highly expedient. The Society is of opinion, that a charge so important might, with greater safety, be confided to an apothecary or dispenser, who had been examined in pharmacy, and had obtained a specific license for the purpose in question. Such substitute for the apprentice would, the Society believes, be most desirable, not less for the comfort and convenience of the practitioner, than for the welfare and security of his patients.

This proposition, if acted upon, might have an additional good effect in terminating the absurd method by which at present the majority of general practitioners seek to be remunerated, viz., by a profit on the medicines they supply.

The foregoing representations suggest the desirableness of obtaining,

1. An improved system of education.
2. A more efficient method of examination.
3. One governing body to preside over the whole profession in England, Scotland, and Ireland.
4. Uniformity of education, and of grade among practitioners.
5. Adequate protection for legally authorised practitioners.
6. The prevention of unqualified persons.
7. The suppression of quackery.
8. The separation of the practitioner and the dispenser in the same individual.
9. The abolition of apprenticeships as at present constituted.
10. The institution of licensed Dispensers.

---

The Society is desirous to submit to the notice of the profession generally, the preceding statement of some of the more prominent evils, which have, for a length of time, weighed heavily on the interests both of the public and of individuals, with a brief outline of the measures calculated, in the opinion of its members, to correct the abuses complained of. As, however, their effectual reformation can be obtained only by the vigorous and united exertions of the whole profession, and not by detached and unconnected efforts, the Society is anxious to invite the co-operation of professional gentlemen in other places, and to recommend to their immediate attention the important questions comprised in this Report. The feeling of the profession at large, as to the defects most urgently requiring amendment, and as to the general principles on which such amendment should be founded, would, in this way, become apparent; and with a view to the settlement of disputed points, the Society would suggest the expediency of a conference being held in London, or elsewhere, composed of deputations sent from different parts of the Empire, whose labours might be directed to the arrangement of a specific plan of Medical Reform, which, if incorporated in a Bill, to be introduced into Parliament, and duly supported by petitions, might be reasonably expected to meet with the consideration of Government and the Legislature.

(Signed) T. E. Headlam, M.D., President.
T. M. Greenhow, Secretary.

*Newcastle-upon-Tyne, November* 27, 1838.

---

### Memorial of the Medical Officers serving on the Bengal Establishment to the Court of Directors of the East India Company.

It is not for us to tell the medical branches of the military and naval services that they have been always treated with little consideration or respect. Look at the army—look at the navy. The officers in each, particularly in the first, enjoy aristocratic connexions and consequently possess parliamentary influence. The results are felt in the tenderness with which the factions of the day approach their rights and privileges. But the zealous, useful, scientific surgeon, who cannot influence votes on a division, is snubbed or actually maltreated, by the heads of his department, and the supreme executive.

The evil can be remedied by union only. Medical men have hitherto proved but a rope of sand, and timidity, or indolence, or despair has prevented any thing like combination. Experience proves that the executive and the legislature equally resist every thing save effective agitation. It is only by rendering the ruling powers uneasy, it is only by the ruled exhibiting their strength, that any boon can be obtained. This is rather a lamentable than a pleasant reflection, but it furnishes such hints for action as cannot well be misconceived.

The case before us, the subject of the present memorial, appears to be a very gross one. It would seem that a "boon" has lately been granted to the officers of the Company's army in India. That boon consists in granting a pension of £365. per annum, to all commissioned officers, whatever their rank, after thirty years' actual service. This boon cannot be considered excessive. What constitutional havoc must thirty years of service in an Indian climate effect. How many must sink in that lengthened period, how few even of the survivors can expect a prolonged enjoyment of the inconsiderable reward! Our readers would not, could not guess, that from this reward, obtained with such delay, purchased at such cost, and likely to be possessed so briefly, the medical officers are excluded! Such is the disgraceful fact. Those men who brave the toils and hazards of military service, in addition to the anxieties and dangers more peculiarly their own—those men are marked out for contumely and injury, and grossly forbidden to participate in the hard-earned fruits of service open to their brother officers! It is impossible to defend this cruel insult, this biting wrong. It is the subject of the Memorial to the Honorable Court of Directors which we subjoin, and if that memorial is ineffectual, if its prayer is disregarded, it will be the most unjust, arrogant, and impolitic exertion of brute power, that any government however constituted could practise in the face of day. We cannot believe it possible that this petition will fail.

### DRAFT OF MEMORIAL.

To the Honourable the Court of Directors of the East India Company,

*The humble MEMORIAL of the Medical Officers serving on the Bengal Establishment,*

Most respectfully Sheweth,

That your Memorialists, in approaching your Honourable Court on this occasion, are impelled by motives of a force and character never before felt by your Medical Service within the experience of its present Members. Your Memorialists refer to their peculiar and unaccountable exclusion from the benefits of the late Boon,—of a specific scale of pensions for periods of service without reference to rank,—which has been conferred on the Officers of the Indian army, in which your Memorialists hold their commissions.

2nd.—Your Memorialists trust that no charge of precipitancy or disposition to hasty or frivolous complaint, can lie against them in this appeal to the justice as well as generosity of your Honourable Court; when it is considered, that the privilege of pension according to length of service has been available to, and extensively enjoyed by, their more fortunate brother Officers during the period of a year and a half past, while many of your Memorialists, the Seniors even of such retiring Officers, are obliged to continue their cheerless toil in this destructive climate, with sinking energies and declining health. It affords evidence equally of the disappointment and deference of your Memorialists, that though the confidence of your Honourable Court's favour extending alike to all departments of the Army, coupled with the absence of specific allusion to the Medical department in your Honourable Court's despatch of the 23rd December 1835, on the subject of the Boon, rendered an appeal by Memorial to the local Government necessary for the ascertainment of the fact of their exclusion or otherwise from its benefits, they have abstained from all direct appeal to your Honourable Court, till they have learned with the deepest concern, that the removal of the present invidious and, your Memorialists trust they may say, unmerited and groundless distinction, is not otherwise to be expected.

3rd.—Your Memorialists beg therefore now to urge with all deference upon the notice of your Honourable Court, that the whole Medical department of India—although some of its Members are occasionally lent for a time to the Civil branch, in like manner as purely Military Officers themselves are sometimes so transferred—is essentially a Military body, suffering all the hardships and privations and dangers of the Military life; and this in addition to the fatigues, anxiety, and deep responsibility—intelligible to few that do not participate therein—of its own peculiar duties in periods of peace as well as war. Their position also as Commissioned Officers of the Army would lead your Memorialists to confidently hope, that it cannot be the ultimate design of the Honourable Court to exclude them from sharing in a Boon of this nature, granted to the Army.

4th.—The essentially Military character of your Medical department cannot be doubted. On the occasion of the late arrangements regarding full and half batta at the different Stations of the Army it was not questioned; your Memorialists, therefore, cannot view otherwise than with surprise and regret the distinction that is attempted, it appears, to be established in regard to the Boon.

5th.—But, independently of the indefeasible identity of the Medical and Military Services giving both an equal claim to such indulgence as that in question, your Memorialists humbly conceive, that there are in their own position circumstances both essential and incidental, that would render the terms of the Boon applicable to the Medical branch in an especial manner. The first of these is the small number of grades in the Medical compared with the purely Military Service, and the consequently longer period required to pass through them, than through the equivalent Military grades, to the higher ranks. In evidence of this we would adduce the fact, that the 10 Bengal Senior Surgeons are men of 30 years' service or upwards, and that these men, in return for such protracted service, if excluded from the operation of the Boon, are entitled to no higher reward than a pension of £191. Nor, by present rules, would they be entitled to more, till they had attained the next higher grade of Superintending Surgeon, (which many of these would not reach in less than seven years), and served therein moreover for the space of two years! Whereas the purely Military Officer of the same period of service, whatever his rank, may retire, under the operation of the present Boon-regulations, on an annual pension of £365. The superiority of the Boon-pension to that of the present rules in the case of these individuals, would indeed be as £450 to £191, taking into consideration the calculated difference of five years' service in favour of Medical Officers, which has ever been allowed, owing to the necessarily later period, by six years, of their admission into the Service. The peculiar hardship of the present Medical Retiring Regulations, as above shewn, cannot fail, it is hoped, to forcibly strike your Honourable Court, and to obtain the concession now so urgently and equitably, they trust, solicited by your Medical servants.

6th.—Another circumstance alluded to above as rendering the indulgence in question peculiarly applicable to the present state of your Medical Service, is the abolition in January and March 1835 of two of the then eleven Bengal Superintending Surgeoncies. Though one of the remaining nine Superintending Surgeons, it is well known, could not even make the tour of the wide field of duty since assigned him, were he to march *every day in the year*, it is of course beyond the province of your Memorialists to discuss the question of state policy or economy which may have dictated the measure in question. They permit themselves indeed no farther remark on the subject beyond the simple statement of the fact, that the attainment of the higher grade,—the point on which Medical pension is at present made dependent,—is thus retarded to nearly twenty-nine thirtieths of Medical Service, to the same degree that promotion would be retarded to those of lower grades among the purely Military Officers by the sudden, abolition of 18 Majorities from the Army-list. It was on men of 30 years' service and upwards that this stoppage of Medical promotion first fell; and upon such also that the consequent retardation operates with greatest force. When to this retardation is added thé singular and anomalous condition to the attainment of the pension of Superintending Surgeon required by present rules, namely two years' actual service in that grade, your Memorialists cannot but confidently hope that your Honourable Court will see herein irresistible reason in favour of extending to the entire Military department the principle, already adopted towards the majority of it, of a specific scale of pension graduated according to actual duration of service and not according to the accident of rank attained.

7th.—Indeed your Honourable Court, when deliberating in what manner a Boon would be best conferred upon your Army, adopted for the first time, the principle of granting pensions according to length of service. Your Memorialists form a part of that Amy, and they hope that they advance no unreasonable pretension, when they urge that the reward for their services should be founded on the same principle. Under this persuasion your Memorialists would here beg to observe, that none of the ten Senior Surgeons mentioned in a preceding paragraph can expect, according to the experience of many years past, to advance through the service of next grade (that of Superintending Surgeon) in a less period than forty years from the date of their entering the Service, by which time, if any should survive, they must be nearly worn out by age and so protracted a sojourn in this climate. Should your Honourable Court be pleased to consider the situation of these individuals as deserving of the remedy for which we now petition, your Memorialists with equal confidence appeal, in forcible support of their prayer, to the case of the Superintending Surgeons, who have no health left to enable them to wait for the increased pension of a Member of the Medical Board, and who are at present denied the pension of £450. a year which has been granted to their Military brethren.

8th.—In addition to what has been above stated, another important but probably little known fact may be urged in favour of the Junior branches, namely— that, according to the rate of promotion from the grade of Lieutenant to that of Captain, and from the grade of Assistant Surgeon to that of Surgeon, calculated by the promotions from the head of each list for the last eight years, the Cadet will on an average reach the Captaincy two years earlier than the cotemporary Assistant Surgeon will attain the Surgeoncy; which, with the difference on entering the Service, gives a total difference of age of eight years between the cotemporary individuals of these parallel ranks. Indeed the obstacles to retirement among the Senior Medical Officers above alluded to, now produce such obstruction to the promotion of the more than 300 members of the Service below them, that this disproportion will be very much increased. In a few years Assistant Surgeons will be found of much more than 20 years' standing: in fact after having served more than 17 years in the country, the period which under existing regulations entitles them to retire on the *pension of their rank*, such pension will be only that of a Lieutenant, or £120. a year! It may perhaps be offered in reply to the above-mentioned difference in age of the parallel Medical and Military grades, that the Assistant Surgeon receives an advantage in pay over his purely Military compeer; but this is a deception; for taking the calculated average of Military and Medical receipts, (the former at 210* rupees a month for the first five years and of 380 rupees a month for the next ten, or till the Captaincy be obtained; and the latter at 450 rupees a month *ab initio* to the period contemplated) it will be found that the Military Officer will on

* These sums are the calculated averages of these grades throughout the Service, all Staff allowances of course included—both Medical and Military.

attaining his Captaincy—say after 15 years' service—have received from the State the sum of Rs. 57,200, while the Medical Officer of the same age—supposing the parties to have entered the Service at the respective ages of 16 and 22—will have received only the sum of Rs. 48,600, or, allowing only five years between the parties, Rs. 54,000; and this disproportion will become greater, as the retarded promotion of the Medical Officer, already pointed out, keeps him longer in the lower grade. It may here be mentioned that it has been found also by a careful calculation, that, during the entire course of Military and Medical service, the actual receipts of the Medical do not exceed those of the Military Officer, who has also rank and honourable distinctions to aspire to altogether unattainable to the former.

9th. Did it appear desirable to strengthen still farther the argument of necessity—already your Memorialists feel more than sufficiently forcible—in favour of the concession solicited from your Honourable Court, they need but advert to the General Orders of the 29th November, 1828, instituting a measure of retrenchment, whereby—apart from all discussion of the principle or question of the necessity—the real fact is, that the two junior thirds of the Medical department were suddenly deprived of 33 per cent. or two-thirds of their then allowances. All the Services suffered severe retrenchment about or subsequent to the period mentioned; but in no way to be compared with that inflicted on the Medical branch.

10th.—Your Memorialists would also beg to draw the attention of your Honourable Court to the recognition and adoption of the principle of pension for service not for rank, that have lately taken place in the Medical department of the Royal Army, where, as in this, it is impossible that all should attain the higher grades. To remove all disheartening circumstances, therefore, tending to weaken Medical men in the efficient discharge of their duty, and with a view to guarantee to every rank a fair and proportionate remuneration, it was determined, that, in lieu of rank and promotion, Surgeons and Assistant Surgeons should be paid and pensioned according to length of service.

11th.—While your Honourable Court has adopted this unvarying and equitable principle towards your Memorialists' brother Officers, and while all the reasons and circumstances above detailed seemed to point *a fortiori* to the necessity and propriety of its application to the Medical branch, your Memorialists depressed and embarrassed, in vain sought for an adequate explanation of their exclusion from this Military Boon. To neither lack of zeal nor to possession of wealth—non-existences both—on the part of your Memorialists as servants of the State, could your Honourable Court's oversight of their deserts and interests in the present case be attributed. In this state of anxious uncertainty, but with the hope of a more satisfactory termination to it, did the rumour reach your Memorialists of your Honourable Court's sanction of the Medical Retiring Fund, affording the explanation that had eluded their research.

12th.—Your Memorialists, it may be guessed, could not for a moment imagine that the Honourable Court considered the slight assistance afforded to the Subscribers of the Medical Retiring Fund, as an equivalent to the Medical Service generally for the loss occasioned by exclusion from the benefits of the Boon, which has been granted to their Regimental brethren; and, in confirmation of this opinion, they beg with all humility and deference to submit the following observations.

13th.—The establishment of a Medical Retiring Fund, although received by your Memorialists with sincere gratitude, can by no means be deemed an equivalent for the scale of pensions now granted to other Officers of the army:—first, because the latter is a free gift, while the former [The Fund] must be purchased and paid for; and secondly, because the Boon from its nature is universally available, while the Fund costs a price that must necessarily and for a long period render it partial and limited in its operation. The Boon granted to our brethren is a free, clear, and unqualified gift available to all; while the favour bestowed in sanctioning the Fund, although the advantages of that institution (still in prospectu) are gratefully acknowledged, must notwithstanding be purchased by Medical Officers individually at such a price, as will necessarily place it beyond the reach of many; for there are unfortunately not a few amongst your Memorialists, who, although anxious to subscribe to it, are yet unable to do so, either from the embarrassment of debt or from the necessity of remitting all personal savings to England for the support of their families. These unfortunate individuals, who are thus reluctantly compelled to forego the advantages of the Retiring Fund, (if excluded from the benefit of the new pension regulations) will find themselves the only Commissioned Officers of the Company's Army, who may serve faithfully for a period of 30 years and upwards, attain an age of from 55 to 60, and be yet only entitled to retire on a pension of £191. per annum.

14th.—Your Memorialists would beg further to remark, that it does not appear that your Honourable Court, although they have sanctioned a new scheme of retirement, have prohibited the establishment of a Retiring Fund among Officers purely Military, should such hereafter be found practicable. Nay, it would appear that encouragement is given to the purchasing out of Military Officers, the principle of the Medical Retiring Fund being thus in practice extended to the Army generally, and probably with more effect, than will ever be exerted by the Medical Retiring Fund in favour of your Memorialists, if a judgment be formed from retirements (two) that have yet taken place through its agency; though it has been currently in operation for two years, and was made retrospectively so for three more. Even in the two instances in which the Fund annuity has been accepted, one only of the retirements can be fairly attributed to its influence. Thus another reason, in the hitherto comparative failure of the Fund, offers itself in favour of the extension to the Medical branch of the Boon granted to the Army.

15th.—The only direct pecuniary aid afforded by your Honourable Court to the Bengal Medical Retiring Fund, is interest at six per cent. on such balance as may from time to time be at its credit in the Government Treasury. This advantage your Memorialists are prepared at once to relinquish, should your Honorable Court think such exaction necessary in extending to them the benefits of the Boon; and they doubt not but that their Medical brethren of the other Presidencies will readily, under the same terms, give up such pecuniary advantages,—in donation, interest and rate of exchange, as your Honourable Court may in their liberality have conferred on them, on the institution of their respective Medical Retiring Funds. Such an arrangement is strongly recommended by its constituting an important step towards establishing that equalization of the circumstances of the Services throughout all the Presidencies, which has professedly been the object and basis of all your late enactments.

16th—In conclusion your Memorialists beg to reiterate their confident reliance on the justice, liberality, candour and consideration of your Honourable Court, in deciding on the prayer herein preferred; and finally to expresss their earnest hope and conviction, that, in the important matter of fixing the length of service that shall entitle Medical Officers to retire on their pensions according to the new scale, due allowance will be made for the late age to which they must have attained, before they can be admitted to the Service; this principle having heretofore been strictly adhered to by your Honourable Court in regulating the relative periods of service for their purely Military and Medical servants. In consideration too of the period of currency of this new scheme of retirement already lost to your Memorialists, they earnestly entreat a speedy as well as favourable answer from your Honourable Court to this their humble but earnest, reasonable, sole and unanimous petition for extension to the Medical branch, with the existing relative terms of service, of the Boon lately conferred on the Military; and your Memorialists as in duty bound shall ever pray.

(Signed) For himself and others whose names are hereunto appended.

To Drs. Forbes and Conolly,

*Editors of the British and Foreign Medical Review, &c.*

Gentlemen,

In No. XIII. page 231, of your Review, is noticed my System of Practical Surgery, part I.;—upon which article I expect that, in your next Number, you will, from a sense of justice, give my remarks.

In his very first paragraph, the self-elected judge pronounces upon the work an unqualified sentence of condemnation; with which may be contrasted the last paragraph, concluding, "*that we cannot recommend it,* (the work) *to our readers on such grounds as these, that it doubtless contains many valuable facts,*" &c.

The intervening part of this effusion is no less entertaining. My book is consigned to oblivion upon the following allegations: the want of definite and indefinite articles; the want of the causes of luxations; the want of *differential diagnosis:* and the want of accuracy in the diction. He has pointed out, I think, three typographical mistakes; for these I am obliged to him; I have myself discovered a few more equally unimportant, but none of them shall be overlooked. The rest of the errors exist only in his own inventive imagination. I should wish an explanation of "*differential diagnosis,*" as well as of the novel doctrine, "that many valuable facts" are no recommendation.

This writer piques himself upon his skill in composition, and his grammatical acumen. Let us test his modest pretensions by the document before us.—It begins, "It is *painful* to us *to be called on* to pass judgment—*as* we are *sorry to say,* we not unfrequently are," &c. "It is painful *to* us to be,"—here is a childish solecism. This unhappy being, indeed, is overwhelmed with pain and sorrow, but what is painful? is it the passing of the judgment? no, it is *the being called on:* what is he sorry for? not the judgment which may injure his neighbour, but, *to say,* is the cause of his affliction! It is desirable to have an explanation of the *as,* for its use here puzzles me as much as the "*differential diagnosis.*" What boy, in his first essay, ever produced so much ungrammatical nonsense in so few words?

Line 15.—"But *as* our time will not allow us to comment upon its whole contents, we *shall* select," &c. The *shall* here reminds me of a London Professor, who wrote to his Scotch friend, "Will you dine with me to-day?" The answer returned was, "Yes I shall!" Might we not have expected better English, even from a north-country adventurer?

Line 26.—"Studying causes *in dislocations of bones.*" Is this an advice to lawyers, or are we to have a new science of etiology? To me certainly the observation is inapplicable, seeing that in my work the causes of dislocations are given in ample detail.

Line 33.—"About flattening of the deltoid muscle:"—these five words exhibit a violation of the common rules of grammar given in every school-book; and the whole proves that our erudite censor knows as much of the use of articles, as of any other part of speech.

This grand specimen of *correct and elegant diction* comprises about five dozen of lines, in which it were not difficult to point out at least an equal number of palpable blunders. It has been said of the author of Hudibras, "that his satirical poem is a burlesque upon poetical language;" but I am not inclined to give my monitor the credit of purposely displaying the faults of which he complains; every intriguing creature is not a Butler.

Any competent person who may take the trouble of "*examining*" the surgery of this sapient critic, will find his literary and surgical accomplishments of equal value; and that to decide upon a system of Surgery, he is a marvellous proper man.

I am, Gentlemen, your obedient Servant,

*Edinburgh,* 33, *York Place,* 21*st January,* 1839. JOHN LIZARS.

To the above letter I received an answer from Dr. Forbes, refusing to insert it without payment, and to which the following was my reply. J. L.

*Edinburgh, 9th Feb.* 1839.

DEAR SIR,—Your answer to me of the 30th ult., is written in a friendly style, though in effect it declares, that, *in your Journal you will publish any tissue of calumny and falsehood, but no vindication.* It has been said, that the corruption and servility of the periodical press are the opprobium of the present age ; I fear, that the Foreign Quarterly will not prove the assertion unfounded. You advise me, no doubt disinterestedly, to suppress my letter, as your Reviewer did, to withdraw my work on surgery, but the greatest ornaments and ablest men of our profession differ from him in opinion. From your offer of inserting for payment my defence in your extra-limites, I infer, that impartiality and truth unbought, find no place within your boundaries ; I *will* therefore, apply where I believe, I *shall* meet with a more favourable reception, and having given you, and your literary English friends, a practical illustration of will and shall,

I am, Dear Sir,

Your very obedient Servant,

JOHN LIZARS.

---

SPECIFIC FOR FEVER, NOW FIRST INTRODUCED INTO EUROPE.

*Address to the Public.*

HAVING, as I perfectly believe,—(and my own convictions are confirmed by the opinions of many experienced Physicians and others, who have witnessed its effects,)—discovered a certain Remedy for Fever, of any type, I feel it to be a duty which I owe to my own character as a professional man, to the Public, and more especially to my Medical brethren, to communicate some particulars relative to this Medicine.

Having ascertained its efficacy in my own practice, my next anxiety was to have the Medicine tried by others, and reported upon. By great exertion, and at considerable expense, I prepared a quantity sufficient for upwards of seven thousand cases, the whole of which I distributed gratuitously into various parts of British, Dutch, and French Guiana, the West Indies, North and South America, Batavia, and the East Indies. The Deputy Inspector General of Army Hospitals in British Guiana, who authorized the use, and officially reported upon the effects, of the Medicine among the Troops in that Colony, also forwarded to the home authorities a numbei of bottles of it, with a recommendation that it should be tried in the Military Hospitals in Malta, Gibraltar, the Ionian Islands, &c. The Reports which I have hitherto received, are uniform in their testimony to the immediate and beneficial effects of the Medicine.

It is my earnest wish, that here, as has already been the case in the West Indies, this Medicine shall rest its claims to public confidence solely on its ascertained merits. I therefore invite the Physicians of the principal Medical Establishments in Great Britain, to the strictest trial of my Fever Drops ; and I have stipulated with MESSRS. PARKER AND COMPANY, King William-street, Charing Cross, that the requisite Medicine for such trials shall be furnished *gratis*, upon proper application.

The form in which I exhibit it, is that of a Tincture. Doses, two ; quantity per dose, two drachms and a half ; interval between doses, three hours. The effects uniformly produced, are, relief of pains in the head, back, and limbs ; slightly narcotic, and highly sudorific. Usual period for total eradication of Fever, from three hours to twenty-four.

*London,* 13*th March,* 1839. CARL WARBURG, M.D.

